# RETRACTED: Dewan et al. The Impact of *Fusobacterium nucleatum* and the Genotypic Biomarker KRAS on Colorectal Cancer Pathogenesis. *Int. J. Mol. Sci.* 2025, *26*, 6958

**DOI:** 10.3390/ijms27167078

**Published:** 2026-08-07

**Authors:** Ahmed Dewan, Ivan Tattoli, Maria Teresa Mascellino

**Affiliations:** 1 College of Life Sciences, Anhui Agriculture University, Hefei 230036, China; ahmeddewan1995@gmail.com; 2 Department of Translational and Precision Medicine, Sapienza University of Rome, 00185 Rome, Italy; ivan.tattoli@uniroma1.it; 3 Department of Public Health and Infectious Diseases, Sapienza University of Rome, 00185 Rome, Italy

The journal retracts the review article titled “The Impact of *Fusobacterium nucleatum* and the Genotypic Biomarker KRAS on Colorectal Cancer Pathogenesis” [1], cited above.

Following publication, concerns were raised regarding the interpretation and presentation of information in figures within this review [1]. 

In accordance with the journal’s standard procedures, the Editorial Office and Editorial Board conducted an investigation. This confirmed that the numerical values presented in Figures 2 and 6 could not be reliably traced to appropriate references cited within the article. During the investigation, the authors were unable to provide sufficient supporting documentation or verifiable source information to confirm the origin and accuracy of the material presented.

As a result, the Editorial Board has lost confidence in the reliability of the article’s content and conclusions and has therefore decided to retract the publication [1], in accordance with the journal’s retraction policy.

This retraction was approved by the Editor-in-Chief of the *International Journal of Molecular Sciences*.

The authors have been informed of this retraction and have indicated their disagreement with this decision.
